# Safety culture in the primary health care settings based on workers with a leadership role: the psychometric properties of the Slovenian-language version of the safety attitudes questionnaire – short form

**DOI:** 10.1186/s12913-018-3594-8

**Published:** 2018-10-11

**Authors:** Zalika Klemenc-Ketis, Irena Makivić, Antonija Poplas-Susič

**Affiliations:** 1 Ljubljana Community Health Centre, Metelkova 9, 1000 Ljubljana, Slovenia; 2 Department of Family Medicine, Faculty of Medicine, University of Maribor, Taborska 8, 2000 Maribor, Slovenia; 3 Department of Family Medicine, Faculty of Medicine, University of Ljubljana, Poljanski nasip 58, 1000 Ljubljana, Slovenia

**Keywords:** Patient safety, Validation studies, Questionnaires

## Abstract

**Background:**

Safety culture describes leader and staff interactions, attitudes, routines, awareness, and practices within an organisation. With this study, we aimed to determine the psychometric properties of the Slovenian-language version of the Safety Attitudes Questionnaire (SAQ) – Short Form in primary health care settings.

**Methods:**

This was a cross-sectional study in the largest primary health care in Slovenia. We invited all employees with a leadership role to participate in the study (*N* = 211). We used the Slovenian-language version of the SAQ – Short Form.

**Results:**

There were 154 participants in the final sample (73.0% response rate), of which 136 (88.3%) were women. The mean age of the sample was 46.2 ± 10.0 years. Exploratory factor analysis put forward six factors: 1) Perceptions of Management; 2) Stress recognition; 3) Teamwork Climate; 4) Communication; 5) Safety Climate; 6) Working Conditions and Satisfaction. This model explained 61.7% of the variance of the safety culture in the primary health care setting. The reliability of the whole scale and of the six factors, assessed using Cronbach’s alpha, was all above 0.78.

**Conclusion:**

The results of our study suggests that the Slovenian-language version of the SAQ – Short Form with six factors could be a reliable and valid tool for measuring the safety culture in the primary health care workers with leadership role In Slovenia. The Slovenian version differed from the original SAQ – Short Form and the majority of other translated versions. Also, the data was from one health centre only and therefore we cannot draw strong conclusions on its external validity.

## Background

Patient safety is an increasingly important part of quality assurance in health care. Within patient safety, a concept of a safety culture has developed which describes leader and staff interactions, attitudes, routines, awareness, and practices within an organisation [1]. As safety culture is a group, and mainly an organisational phenomenon, it differs across the different levels and organisations of health care [2, 3], so appropriate tools should be employed in order to capture the true state of affairs. Safety culture differs from safety climate the latter referring to the measurable components of safety culture, a kind of a snapshot of safety culture at the given moment in time [4].

Several tools have been developed to measure the safety attitudes of healthcare providers [5–7]. Recent reviews of the Safety Climate Surveys in health care settings showed up to 17 [7, 8] available surveys, however only three were developed for a primary health care settings [7, 8]. Another systematic review on tools for safety culture in primary care [6] put forward the Safety Attitudes Questionnaire (SAQ) [9] as one of the widely used and also one of the most appropriate instruments for assessing patient safety culture. In 2007, the SAQ was adapted to outpatient (primary healthcare) settings [5]. This adapted version, the SAQ – Ambulatory Version (SAQ-AV), was found to be a reliable tool for comparing attitudes across different professional groups of healthcare providers outside hospitals [5, 10, 11]. Its shorter version, the SAO Short Form is being increasingly used in many countries [9, 12–18].

In Slovenia, quality and safety at the primary health care level have been the focus of study over the past several years [19–22]. Patient safety features in primary healthcare have been investigated through a study on the Quality and Costs of Primary Care in Europe (QUALICOPC), dealing with the organisation and accessibility of primary healthcare services [23]. In addition, safety culture has been measured by the Slovenian-language version of the SAQ-AV in out-of-hours primary health care settings [24–26]. Out-of-hours settings in Slovenia differ from primary healthcare centres in terms of organisation, location, and staff. Out-of-hours health services are combined with emergency medical services and are available both at the same place and time. Sometimes, they are located in hospitals and sometimes in the healthcare centres. Professionals that work in OOHC are family physicians, emergency physicians, and emergency nurses. Sometimes, there are also laboratory technicians and radiology technicians. Usually, emergency nurses work only in OOHC settings while family physicians work in their practice and in OOHC on the basis of rotation. An out-of-hours health centre has thus a unified leadership and can be seen as a “natural social unit”; which is a validation criteria for organizational climate measurements [27].

To date, the safety culture in primary health care settings such as primary health care centres has not been measured in Slovenia. Also, the SAQ – Short Form has not yet been validated in the Slovenian language.

Within this study we wanted to fulfil the following aims: 1) to test the reliability and validity of the Slovenian-language version of SAQ – Short Form in primary health care settings, and 2) to determine the factor structure of the Slovenian-language version of SAQ– Short Form in primary health care settings.

## Methods

### Research design and setting

We performed a cross-sectional study in the largest community health centre in Slovenia. This health centre provides healthcare services for the municipality of Ljubljana, which comprises around 280,000 people. It is divided into eight units, which are located in separate buildings in different parts of Ljubljana. It employs around 1500 employees of different medical and non-medical backgrounds.

### Participants

We invited all the employees with a leadership role to participate in the study (*N* = 211). Employees with a leadership role come from different professional backgrounds (i.e. physicians, dentists, registered nurses, nurse assistants, administrative staff etc.). They are appointed to be leaders of different units within the health centre, such as chief of nurses, chief of physicians, chief of whole units, director of health centre etc. They work mostly within their professional fields, but have a certain amount of their working time dedicated to their leadership tasks. We thought that a homogenous group such as workers with a leadership role would be appropriate for a validation study as they are the ones responsible for the safety culture and their role in creating a proper safety environment is crucial.

### Measures

We used the SAQ – Short Form [9] which consists of 36 items. Each item of the questionnaire is answered on a 5-point Likert scale by which the respondents indicate their level of agreement with the statement ranging from “disagree strongly” to “agree strongly”). In the analysis, the scores of negatively worded items were reversed so that higher scores always indicated a more positive evaluation of the safety culture. There are six factors in the original SAQ – Short Form: Teamwork Climate (items 1–6), Safety Climate (items 7–13), Job Satisfaction (items 15–19) Stress Recognition (items 20–23), Perceptions of Management (items 24–28), and Working Conditions (items 29–32). Items 14 and 33–36 are not included in any of the factors.

We were granted permission to use this questionnaire by the University of Texas at Houston-Memorial Hermann, Center for Health care Quality and Safety. The permission was given on June 3, 2016.

We also collected data on demographic characteristics (gender, age, role, work experience, working hours, and location of work).

### Data collection

The data was collected through an electronic survey. The link to the survey was sent to the email addresses of the participants in February 2017. The first reminder was sent after two weeks, and the second two weeks after the first. Participation was confidential, possible identifiers such as e-mail and IP addresses were removed by the administrative coordinator in the project. It was not possible for the researchers to link the participants to their responses.

### Statistical analysis

#### Variability of the data set

We determined the skewness and kurtosis of the data set. Skewness was 0.210 and kurtosis was 0.332. We concluded that for a given sample size, the measures of the variability indicated a normal distribution of the data [28].

#### Confirmatory factor analysis

We performed a confirmatory factor analysis (CFA) of the SAQ – Short Form. We used the items that were part of the original factors (31 items). The following indices for the good CFA model fit were considered: 1) the chi-square goodness-of-fit: the model is acceptable if the *p*-value of chi-square is not significant, 2) relative chi-square, which is the chi-square divided by degrees of freedom (df), should range up to 3 [29]; 3) Comparative Fit Index (CFI) should lie within 0.90–1.00 for a fit model; 4) the Normed Fit Index (NFI) should range between 0 and 1 with a value of 0.90 or greater indicating of a good model fit; and 5) Root Mean Square Error of Approximation (RMSEA): a value of about 0.05 or less would indicate a close fit of the model in relation to df [30].

#### Exploratory factor analysis

Our CFA model did not provide a good fit and therefore the explorative factor analysis (EFA) was carried out with an Oblimin rotation (with Kaiser normalisation) on 31 items as in the original factor model [9]. As the results was not acceptable, we decided to perform the EFA on all 36 items in the questionnaire. This yielded an acceptable result with good share of explained variance. A cut-off point for factors loadings was 0.300. We also performed a Kaiser-Meyer-Olkin analysis and a Bartlett test (both measures test how suited is the data for factor analysis). We determined that the Kaiser-Meyer-Olkin measure should be < 0.8 and that the Bartlett test should be < 0.001 [31]. For each of the six factors, the scale scores were calculated by obtaining the mean of the item scores within one factor. Next, correlations between the scale scores were calculated to determine construct validity. Reliability (internal consistency) was assessed using the Cronbach’s alpha. Values of Cronbach’s alpha over 0.7 are considered acceptable, over 0.8 good, and over 0.9 excellent [32].

## Results

### Sample characteristics

There were 154 participants in the final sample (73.0% response rate), of which 136 (88.3%) were women. Most of the sample consisted of physicians and registere nurses. All health centres’ units were represented in the sample (Table 1). The mean age of the sample was 46.2 ± 10.0 years; the mean time in current post was 13.6 ± 10.0 years, and the mean number of weekly working hours was 36.2 ± 10.4. The mean number of years of clinical experiences participants was 21.9 ± 10.1 years.

**Table 1:** Demographic characteristics of the participants

Characteristic	Number (%)
Gender	
Female Male	136 (88.3)18 (11.7)
Profile	
Physician, dentist	54 (35.1)
Registered nurse	36 (23.4)
Manager	28 (18.2)
Nurse assistant	18 (11.7)
Other clinical staff	16 (10.4)
Administrative staff	2 (1.3)
Community Health Centre unit	
Unit 1	41 (26.6)
Unit 2	24 (15.6)
Unit 3	23 (14.9)
Unit 4	19 (12.3)
Unit 5	19 (12.3)
Unit 6	16 (10.4)
Unit 7	8 (5.2)
Unit 8	4 (2.6)

### Confirmatory factor analysis

CFA model on the original six factors with the appropriate items (see Methods section) did not provide a good model fit on all test. *P* value was < 0.001, the relative chi-square was 1.636, CFI was 0.874, NFI was 0.737, and RMSEA was 0.064.

### Factor structure

The on 36 items put forward six factors: 1) Perceptions of Management; 2) Stress recognition; 3) Teamwork Climate; 4) Communication; 5) Safety Climate; 6) Working Conditions and Satisfaction (Table 2). This model explained 61.7% of the variance. The Kaiser-Meyer-Olkin measure was 0.824 and the Bartlett test was significant (*p* < 0.001).

**Table 2:** Factor model and reliability

Item	Factor loading	Cronbach’s alpha	Cronbach’s alpha if item deleted
Factor 1: Perception of Management		0.869	
Management is doing a good job.	0.837		0.847
Management doesn’t knowingly compromise patient safety.	0.814		0.853
Management supports my daily efforts.	0.808		0.850
I get adequate and timely information about events that might affect my work.	0.737		0.854
All the necessary information for diagnostic and therapeutic decisions is routinely available to me.	0.732		0.862
Nurse input is well received in this clinical area.	0.706		0.855
My suggestions about safety would be acted upon if I expressed them to management.	0.678		0.855
Problem personnel are dealt with constructively.	0.610		0.858
I know the proper channels to direct questions regarding patient safety in this clinical area.	0.604		0.862
I am encouraged by my colleagues to report any patient safety concerns I may have.	0.594		0.858
The culture in this clinical area makes it easy to learn from the errors of others.	0.394		0.867
The levels of staffing in this clinical area are sufficient to handle the number of patients.	0.356		0.883
Factor 2: Stress Recognition		0.862	
I am less effective at work when fatigued.	0.883		0.789
I am more likely to make errors in tense or hostile situations.	0.812		0.833
When my workload becomes excessive, my performance is impaired.	0.779		0.807
Fatigue impairs my performance during emergency situations (e.g. emergency resuscitation, seizure).	0.636		0.866
Factor 3: Teamwork Climate		0.870	
I experience good collaboration with staff physicians in this clinical area.	−0.825		0.791
I experience good collaboration with nurses in this clinical area.	−0.739		0.823
I experience good collaboration with pharmacists in this clinical area.	−0.637		0.850
The physicians and nurses here work together as a well-coordinated team.	−0.591		0.868
Factor 4: Communication		0.830	
In this clinical area it is difficult to discuss errors.	0.825		0.782
Communication breakdowns that lead to delays in delivery of care are common.	0.739		0.795
Disagreements in this clinical area are resolved appropriately.	0.673		0.799
In this clinical area it is difficult to speak up if I perceive a problem with patient care.	0.643		0.787
I have the support I need from other personnel to care for patients.	0.593		0.824
This hospital does a good job of training new personnel.	0.568		0.823
Factor 5: Safety Climate		0.781	
Medical errors are handled appropriately in this clinical area.	0.813		0.664
I receive appropriate feedback about my performance.	0.799		0.786
I would feel safe being treated here as a patient.	0.666		0.707
It is easy for personnel here to ask questions when there is something that they do not understand.	0.610		0.747
Factor 6: Working Conditions and Satisfaction		0.874	
This is a good place to work.	−0.926		0.831
I am proud to work in this clinical area.	−0.912		0.824
Working here is like being part of a large family.	−0.825		0.837
Morale in this clinical area is high.	−0.713		0.850
I like my job.	−0.589		0.880
Trainees in my discipline are adequately supervised.	−0.564		0.880

There was a significant difference in items belonging to the factors of the original scale and of our scale. Except for the factor Stress Recognition, all factors in our factor model had partially or completely different items (Table 3).

**Table 3:** The distribution of items within factors on the original SAQ – Short Form scale and on our SAQ – Short Form scale

Scale	Perceptions of Management	Stress Recognition	Teamwork Climate	Communication	Safety Climate	Working conditions and Satisfaction
SAQ – Short Form (original)	Items 24–28	Items 20–23	Items 1–6	/	Items 7–13	/
SAQ – Short Form (our study)	Items 1, 9, 12–14, 24–29, 31	Items 20–23	Items 6, 33–35	Items 2–4, 11, 30, 36	Items 5, 7, 8, 10	Items 15–19, 32

### Reliability of the SAQ – Short form and its factors

The reliability of the whole scale, through Cronbach’s alpha, was 0.963 and the reliability of the individual factors was also good (Table 2).

### Construct validity

All the factors except Stress recognition were significantly correlated with each other (Table 4), while Stress recognition was correlated just with the Communication.

**Table 4:** Mean scores, standard deviations and intercorrelations of the five factors

Factor	Mean	SD	F1	F2	F3	F4	F5
Perceptions of Management (F1)	3.68	0.496					
Stress recognition (F2)	3.50	0.893	0.012				
Teamwork Climate (F3)	4.12	0.586	0.572^a^	−0.026			
Communication (F4)	3.84	0.566	0.621^a^	−0.238^b^	0.638^a^		
Safety Climate (F5)	4.15	0.571	0.645^a^	−0.183	0.605^a^	0.658^a^	
Working Conditions and Satisfaction (F6)	4.07	0.600	0.617^a^	−0.040	0.687^a^	0.567^a^	0.533^a^

^a^Correlation is significant at the 0.01 level

^b^Correlation is significant at the 0.05 level

## Discussion

The Slovenian-language version of the SAQ – Short Form used with the employees of the largest primary health care centre in Slovenia did not fit perfectly to the original factor structure [9]. Our new factor model revealed six factors: 1) Perceptions of Management; 2) Stress recognition; 3) Teamwork Climate; 4) Communication; 5) Safety Climate; 6) Working Conditions and Satisfaction. Cronbach’s alphas of the factors were good, indicating no problems with the factors. The six-factor model of the Slovenian SAQ – Short Form covered 36 items.

Our factor structure differs from the original one [9] in the context of some factors. Factors Teamwork Climate, Perceptions of Management, Working Conditions, Safety Climate, and Stress Recognition are the same as in the original factor structure [9]. However, the items belonging to these factors differ (see Table 3). Our model also revealed one new factor (Communication) and one partly new factor (Working Conditions and Satisfaction). The latter is actually a combination of two factors from the original SAQ – Short Form scale. The factor Communication has not been revealed within the original scale but has already been revealed as an individual factor of the Slovenian-language version of the SAQ-AV [25]. Communication has also been recognised in other safety culture measurement tools as an important domain within the safety climate or culture [7, 10, 33].

Some items have negative loadings (all items in factors Teamwork Climate and Working Conditions and Satisfaction). This indicates that these factors are negatively correlated to the construct. It is a general agreement that the strength of a loading needs to be measured by its absolute value with the possibility that preconceptions about appropriate reverse coding might need modification based on the signs of factor loadings [34]. Therefore, we decided to keep the original wording of the items.

All the factors measured the same construct except for Stress Recognition. This could be due to the fact that other factors related more to work and the working environment, and Stress Recognition more to personality traits. Stress Recognition was weakly correlated with Communication, which indicates that if the working environment stimulates communication about safety issues, the workers feel that they manage stress better.

The validation studies on SAQ – Short Form in Norwegian ([35], Italian [13], Portuguese [14], Chinese [15], Swedish [16, 17], and German [18] language confirmed the original factor structure. However, they were carried out in the secondary health care level which makes it difficult to compare the results of our study to them as our study was done in primary health care settings. Our result of a CFA not perfectly fitting to the original factor model indicates that there are differences in the safety culture between primary and secondary health care levels which has already been pointed out elsewhere [6, 33]. Namely, primary health care differs from hospitals in terms of organisational structure, administrative and clinical processes and the reasons for encounters [36]. However, it could also indicate that the tool used (the SAQ – Short Form) was not specific enough for the primary health care settings and another tool could be used.

The safety climate and culture research in health care in general has been predominately conducted within acute care settings and hospital settings [7] therefore the existing measurements in primary health care settings are relatively new which makes our study an important one for this field of research.

The differences in factor structures between countries make cross-country comparisons of patient safety culture challenging. The structural differences may reflect cross-national variation in the nature and structure of primary care, or it may mean that item wordings trigger different connotations in different languages. For these reasons a wise option might be to compare countries at the item level rather than at the factor level [10].

The differences in the safety culture between the different levels of health care require different tools for measurements that would be developed for primary care or hospital safety culture measurement tools adapted for the use at the primary health care level. Since there is currently no consensus on which tool is the best to use [7], further research should focus on performing the \adaptations and validations of the existing tools for use at the primary health care level, with special emphasis on establishing the construct and criterion validity [7]. Another option would also be a development of a new scale which would be based on the theoretical model of the safety culture at the primary health care level.

Our study has some limitations that have to be mentioned. We are aware of the fact that an EFA always produces a solution, but does not assess the risk that the EFA solution only describes the data set, and it may not be possible to generalise. Due to this fact, the results of our study may not be generalised to the whole population. The sample size in our study was limited to employees with a leadership role, which could produce a selection bias and therefore limits the validity of our results. However, the results were very similar to our previous study [25] and also to the Dutch study [10] (both performed at the primary health care level) and this gives us confidence in the validity of our results. Although the response rate was high, there is no information on the characteristics of the non-respondents, so this could also be a source of bias.

We used the SAQ – Short Form for the primary health care settings despite the fact that there are other specific primary health care tools available [6, 7] and might have been a better tool for our study. This is another limitation of our study.

## Conclusions

The results of our study suggests that the Slovenian-language version of the SAQ – Short Form with six factors could be a reliable and valid tool for measuring the safety culture in the primary health care workers with leadership role. The factors and item loadings in our study differed from the original SAQ – Short Form and the majority of other translated versions.

Further studies should explore the safety culture of other primary health care organisations, and perhaps also use other tools for measuring it in order to recognise other factors that are also important. Possibly, another tool for a more comprehensive measurement of safety culture that would be based in the theoretical frameworks should be developed.

## Abbreviations

CFA: Confirmatory factor analysis
CFI: Comparative Fit Index
df: Degree of freedom
EFA: Exploratory factor analysis
IP: Internet Protocol
NFI: The Normed Fit Index
QUALICOPC: Quality and Costs of Primary Care in Europe
RMSEA: Root Mean Square Error of Approximation
SAQ: Safety Attitudes Questionnaire
SAQ-AV: Safety Attitudes Questionnaire-ambulatory version
